# An ImageJ‐based tool for three‐dimensional registration between different types of microscopic images

**DOI:** 10.1111/dgd.12835

**Published:** 2023-01-14

**Authors:** Hiroshi Koyama, Kanae Kishi, Seiya Mikoshiba, Toshihiko Fujimori

**Affiliations:** ^1^ Division of Embryology National Institute for Basic Biology Okazaki Japan; ^2^ Department of Basic Biology, School of Life Science SOKENDAI (The Graduate University for Advanced Studies) Okazaki Japan; ^3^ Japan Science and Technology Agency, Core Research for Evolutional Science and Technology (JST‐CREST) Kawaguchi Japan; ^4^ Graduate School of Science Nagoya University Nagoya Japan

**Keywords:** image registration, ImageJ, mouse early embryo, three‐dimensional image rotation

## Abstract

Three‐dimensional (3D) registration (i.e., alignment) between two microscopic images is very helpful to study tissues that do not adhere to substrates, such as mouse embryos and organoids, which are often 3D rotated during imaging. However, there is no 3D registration tool easily accessible for experimental biologists. Here we developed an ImageJ‐based tool which allows for 3D registration accompanied with both quantitative evaluation of the accuracy and reconstruction of 3D rotated images. In this tool, several landmarks are manually provided in two images to be aligned, and 3D rotation is computed so that the distances between the paired landmarks from the two images are minimized. By simultaneously providing multiple points (e.g., all nuclei in the regions of interest) other than the landmarks in the two images, the correspondence of each point between the two images, i.e., to which nucleus in one image a certain nucleus in another image corresponds, is quantitatively explored. Furthermore, 3D rotation is applied to one of the two images, resulting in reconstruction of 3D rotated images. We demonstrated that this tool successfully achieved 3D registration and reconstruction of images in mouse pre‐ and post‐implantation embryos, where one image was obtained during live imaging and another image was obtained from fixed embryos after live imaging. This approach provides a versatile tool applicable for various tissues and species.

## Graphical abstract

We propose a user‐friendly image processing method by which we can three‐dimensionally align two images with multiple *z*‐slices. This method enables us to easily compare two tissues or embryos labeled by different markers under three‐dimensional situations.

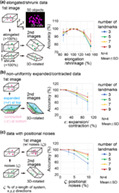

## 1 INTRODUCTION

**FIGURE 1:**
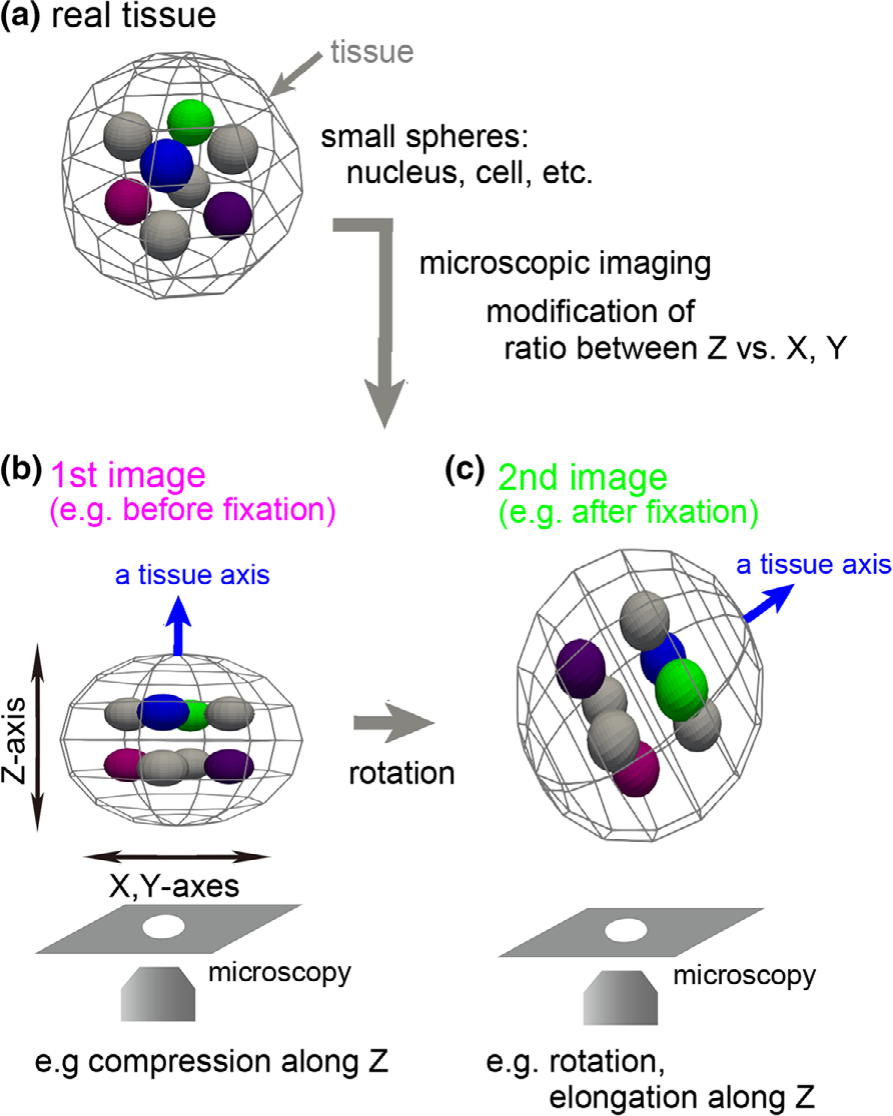
Illustration of rotation and distortion of a specimen during preparation. (a) A tissue is illustrated with inner objects. In this case, the tissue and the inner objects are depicted as spheres. (b) An example of image acquisition of a tissue. The acquired image can be shrunk or elongated along the *z*‐axis. (c) A rotated tissue. During experimental procedures including fixation, the tissue may be rotated (“a tissue axis” between (b) and (c))

Three‐dimensional (3D) imaging is a central technique in developmental biology and organoid studies, which is achieved by confocal microscopy, multiphoton microscopy, micro‐computed tomography (CT), etc. During live imaging of tissues such as early embryos of mice, chordates, and echinoderms and organoids, they can be 3D rotated in the cultured medium/liquid because they do not adhere to substrates. In studies related to cell tracking and cell lineages, researchers have to make much effort to determine the correspondence of each cell between images before and after the rotation (Koyama et al., 2022; Kurotaki et al., 2007; Pokrass et al., 2020; Simon et al., 2020). This includes cases where researchers compare live imaging data with images obtained from samples fixed after live imaging (Figure 1) (Pokrass et al., 2020; Simon et al., 2020); for example, characteristics different from those obtained from the live imaging are visualized from fixed samples that were subjected to immunostaining, while the tissues are rotated during fixation. In addition, alignment between different embryos at a similar embryonic stage is also helpful for comparing differences in cell lineage and position between the different embryos (Onuma et al., 2020). Under these situations, researchers physically correct the rotations during sample preparation on microscopic stages or correct on the basis of manual image processing (Onuma et al., 2020; Pokrass & Regot, 2021; Simon et al., 2020), both of which are usually time‐consuming processes. In the former case, an exact correction is almost impossible (i.e., spatial discrepancies between two images remain to some extent), which may be problematic for spatially intricate regions in tissues. In the latter case, the correspondence of each cell between two images is determined without reconstructing 3D rotated images, and thus the outcomes of the operations are not clearly presented. Therefore, it is difficult for other researchers (and even for the researchers doing the manual operations) to evaluate whether the corrections are reliable. To improve these situations, it is critical to develop an image processing tool for 3D registration accompanied with both visualization and quantitative evaluation of the outcomes, which should be easily accessible for experimental biologists who are not so familiar with image processing.

For experimental biologists, ImageJ (https://imagej.nih.gov/ij/) (Schneider et al., 2012) and its high‐functionality version Fiji (https://imagej.net/software/fiji/) (Schindelin et al., 2012) are the most widely accessible image processing tools. In default plugins implemented in Fiji, “Correct 3D drift” is a 3D registration tool, but it only considers 3D translation (i.e., *x*‐, *y*‐, and *z*‐directional movements) but not rotation (Parslow et al., 2014). Thus, basically existing methods rely on the extension of two‐dimensional (2D) registration, and a genuine 3D orientation method is not yet available. Another 3D registration plugin called “Descriptor‐based registration (2d/3d)” was developed for registration of images obtained from selective plane illumination microscopy. This tool is implemented for specific situations where many beads are embedded as landmarks in samples and are computationally detected for subsequent usage of 3D registration (Preibisch et al., 2010). A more primitive way is manual operations of the “Rotate” and “Reslice” tools, both of which are basic functions in ImageJ/Fiji; through multiple cycles of these two tools, any 3D rotation and subsequent 3D reconstruction can in principle be achieved. However, as far as we tried, it is very hard to determine correct 3D rotations and angles of reslices, probably except for researchers who can easily imagine 3D rotation of objects. To facilitate image analysis, there is an urgent need for an efficient user‐friendly tool. Image processing tools other than ImageJ have been developed for experimental biologists, especially for segmentation of 2D epithelial cells and subsequent quantitative analyses (Heller et al., 2016; Tan et al., 2021) and for segmentation of objects in 3D tissues (Azuma & Onami, 2017; Bao et al., 2006; McDole et al., 2018). However, no practical solution for 3D registration considering rotation is publicly available for experimental biologists.

In the present study, we developed a versatile 3D registration/rotation tool which is applicable for many types of 3D images and can be run in ImageJ. To ensure versatility, our method is based on registration of multiple points which are arbitrarily defined in two images.

## 2 MATERIALS AND METHODS

### 2.1 Mouse embryos

**FIGURE 2:**
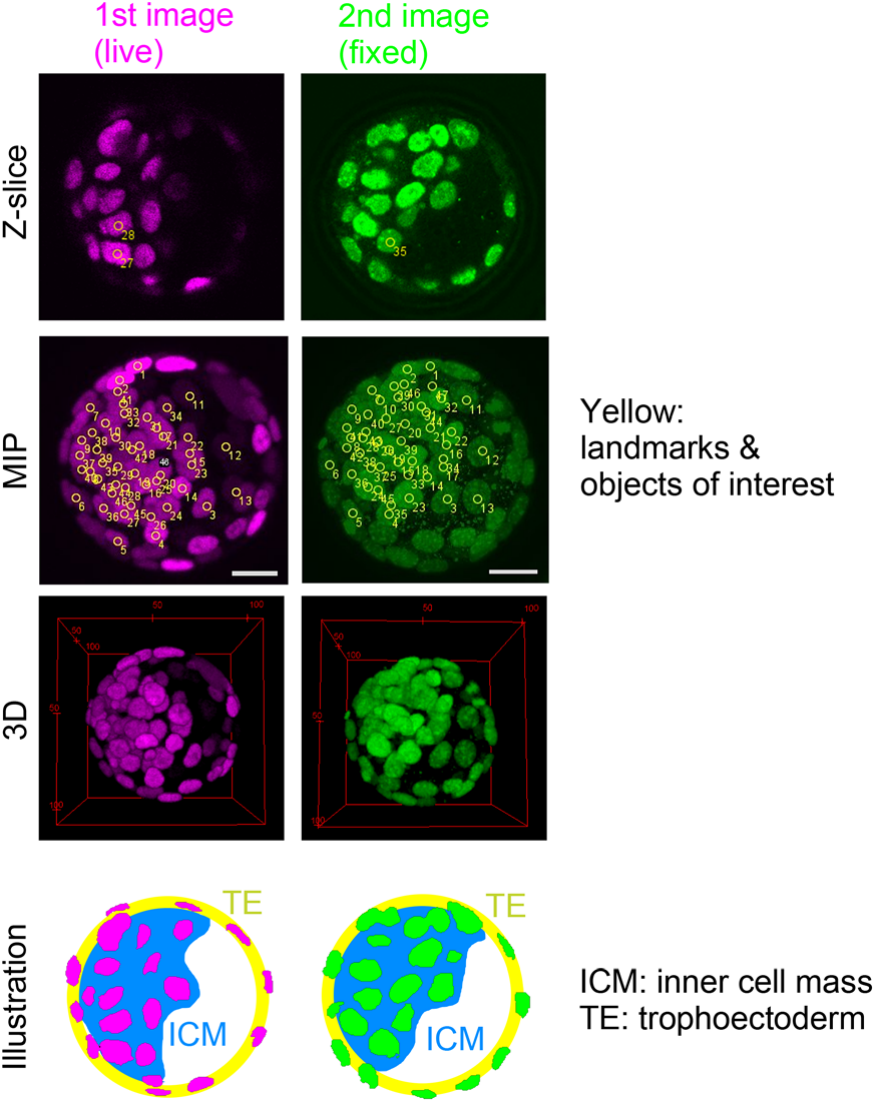
Rotated image of a mouse blastocyst. The first and second images are acquired from a live or fixed embryo. A *z*‐slice, maximum intensity projection (MIP), and three‐dimensional (3D) view of the images are shown. The regions of the inner cell mass (ICM) are illustrated for each image. The labeling of landmarks and objects is described in Appendix A. Scale bars = 20 μm

Mouse embryos at the blastocyst stage were mainly used for testing. We performed confocal fluorescence microscopic imaging of fluorescently labeled nuclei in living embryos in a manner similar to our previous work (Figure 2, first image) (Azuma & Onami, 2017; Koyama et al., 2022). Subsequently, we fixed the embryos, stained the nuclei by Hoechst, and then imaged them (Figure 2, second image). The blastocysts are composed of two cell types: the trophectoderm (TE) cells form an outermost layer and the inner cell mass (ICM) cells form an inner cell aggregate with a high cell density (Figure 2, illustration). The images obtained from the fixed embryos showed rotation compared with the images obtained from living embryos (Figure 2; see the location of ICMs).

The microscopic imaging conditions for the blastocysts were as follows: confocal microscopy was conducted (A1 laser scanning confocal microscope, Nikon, Japan) with a 60× objective (PlanApo; WI; NA = 1.20, Nikon, Japan), and *z*‐slices were separated by 0.575 μm for live embryos and by 0.625 μm for fixed embryos. The nuclei in the live or fixed embryos were labeled by YFP conjugated with a nuclear localization signal or Hoechst, respectively.

We also used mouse post‐implantation embryos as another test case (5.5 days after fertilization). The nuclei before and after fixation were visualized similarly to the case of the blastocysts. In addition, we stained F‐actin by phalloidin (Phalloidin‐Alexa647; Molecular Probes, A22287, Oregon) in the fixed embryos. The microscopic imaging conditions were the same as those for the blastocyst imaging experiments, except for the objective (PlanApo; 40×; dry; NA = 0.95, Nikon, Japan) and the spacing between *z*‐slices (1.0 μm).

### 2.2 Overview of methods for 3D registration

**FIGURE 3:**
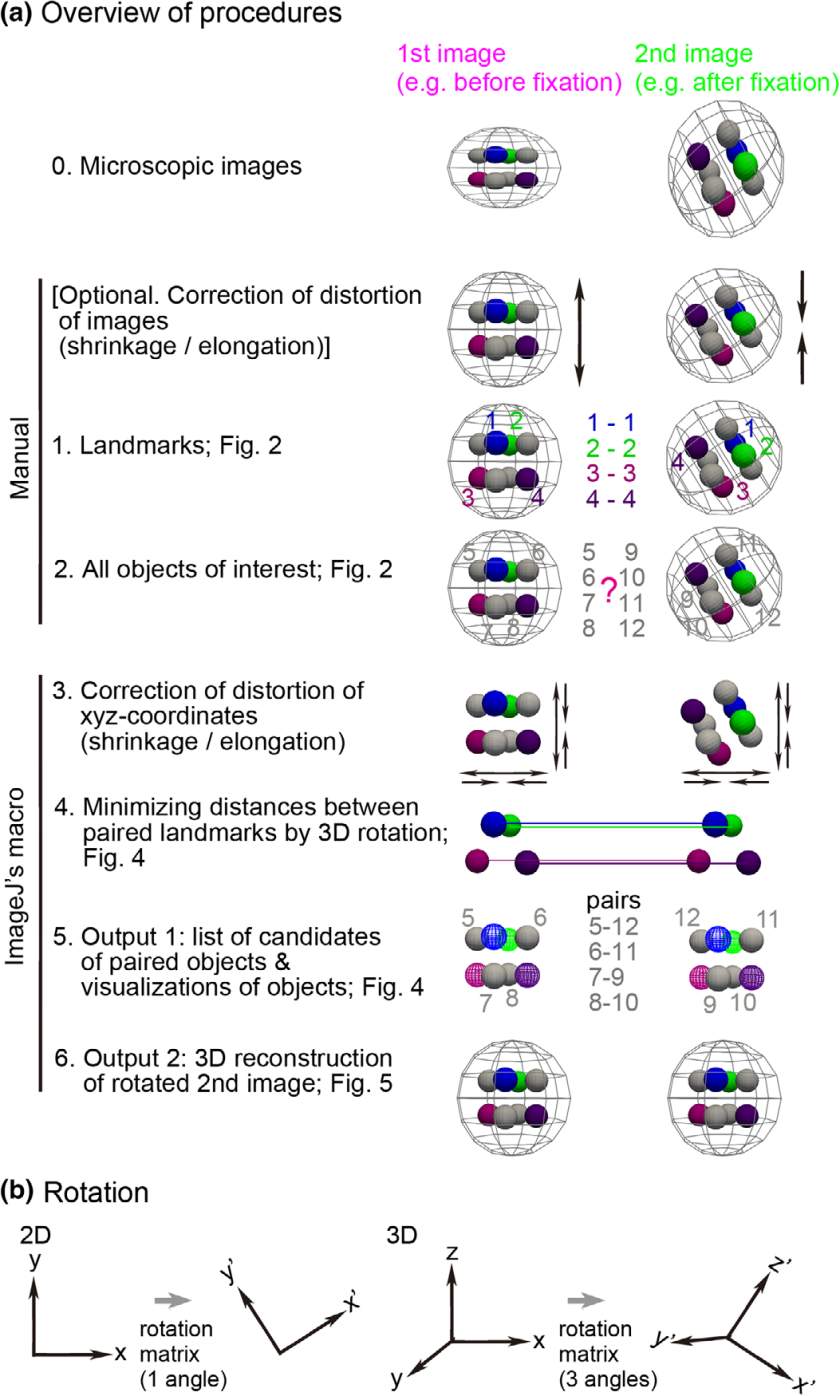
Procedures of three‐dimensional (3D) registration and reconstruction. (a) The procedures of our method are illustrated. At step 0, microscopic images are shown where the second image is rotated compared with the first image. At the “Optional” step, the shrinkage or elongation of the two images is corrected (arrows). At step 1, four landmarks are shown (#1–4). At step 2, objects of interest are labeled by non‐overlapped numbers between the two images (#5–8 vs. #9–12). At step 3, shrinkage or elongation of the *xyz*‐coordinates of the landmarks and the objects of interest is corrected. If shrinkage or elongation of the images has already been corrected at the “Optional” step, step 3 is not required. At step 4, the landmarks in the second image are optimally rotated. At step 5, the paired objects are identified (e.g., 5–12, 6–11). At step 6, the second image is rotated to be aligned with the first image, and the rotated image is reconstructed. (b) Definition of 3D rotation. In the case of two‐dimensional (2D) rotation, the rotation matrix contains one angle (*θ* in Appendix A). In the case of 3D rotation, the rotation matrix contains three angles (*ϕ*, *θ*, and *ψ* in Appendix A)

For cell lineage studies in mouse early embryos (Kurotaki et al., 2007; Pokrass et al., 2020; Simon et al., 2020), 3D registration during live imaging or between live and fixed embryos substantially supports the analyses. We developed a landmark‐based 3D registration method and applied it to blastocysts. The method is illustrated in Figure 3. In the first step, as landmarks, we manually chose several pairs of the same nuclei between the first and second images (Figure 3(a), step 1). In addition to the landmarks, we manually chose all objects (i.e., nuclei) of interest in both images, which are not paired at this moment (Figure 3(a), step 2). The manual steps needed in our method are limited to the above two steps, and thus user effort is minimal. The next step is the core of our method, where 3D rotation is computationally performed so that the sum of the distances between the paired landmarks, i.e., the cost function, is minimized (Figure 3(a), step 4). In the present case, the landmarks in the second image were rotated. In general, 3D rotation is expressed as a matrix composed of three rotational angles (Figure 3(b)), while 2D rotation is expressed as a matrix composed of one rotational angle (Figure 3(b)). Therefore, the optimal values of these three angles were computed; the mathematical algorithm is presented in Appendix A.

By using the optimal values of the three angles, we performed the following two analyses. (1) The *xyz*‐coordinates of the nuclei of interest chosen in the previous step were computationally rotated according to the three rotational angles (Figure 3(a), step 5). For each nucleus in the live embryos, we computationally determined the nearest nucleus in the fixed embryos. In other words, we determined the correspondences of the several tens of nuclei between the live and fixed embryos (Figure 3(a), step 5, “pairs”). (2) We also applied the rotation based on the three rotational angles to the images from the fixed embryos, and reconstructed 3D images (Figure 3(a), step 6). Consequently, we can easily compare the resultant 3D images with the images obtained from the live embryos. These two analyses enabled us to quantitatively and visually determine the correspondences of the nuclei between the live and fixed embryos.

In addition to the above steps, we implemented optional steps to adapt to real situations. In real tissues, fixation often shrinks tissues. Moreover, microscopic imaging conditions cause shrinkage or elongation of 3D images along the *z*‐axis, for example, due to differences in refractive index between the glass of the glass‐base dishes and the medium. Severe shrinkage or elongation can spoil the 3D registration. We can revise the *x*‐, *y*‐, and *z*‐scales in both the live and fixed embryos before or after the choice of the landmarks (Figure 3(a), “[Optional…” and step 3).

## 3 RESULTS

### 3.1 3D rotation of landmarks and objects of interest

**FIGURE 4:**
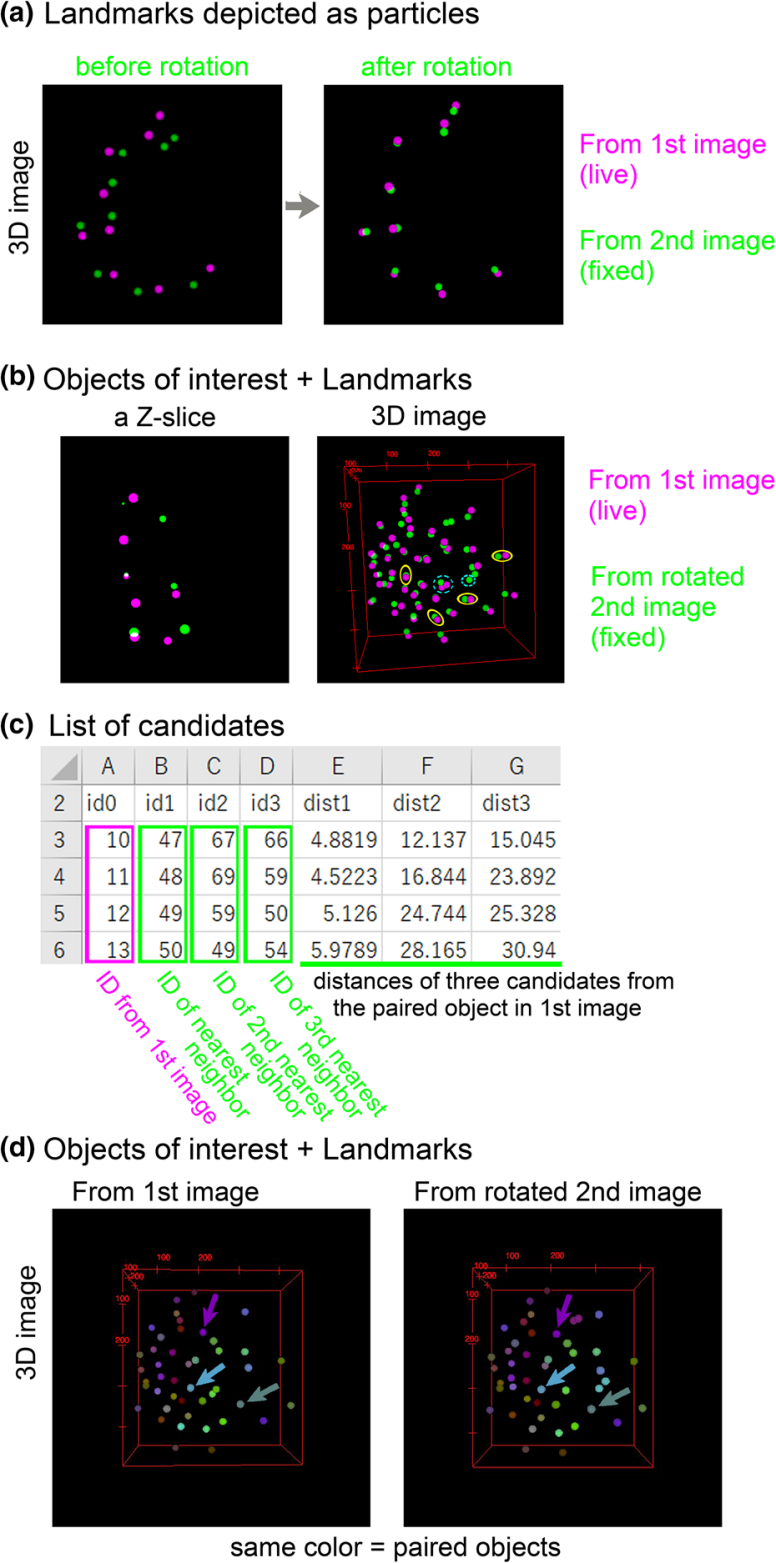
Registration of landmarks and objects of interest. (a) Landmarks in the first and second images are depicted as particles in three‐dimensional (3D) images. Images before and after the rotation of the second image are shown. The 3D images were generated by using Fiji > Plugins > 3D Viewer; all 3D images in this article were generated by the 3D Viewer. (b) Objects of interest in the first and second images are depicted as particles in 3D images. The landmarks are also depicted. Yellow circles, some examples of paired objects; light blue circles with dashed lines, a few examples of unsuccessfully paired objects. (c) Quantitative evaluation of pairing. For each object of interest in the first image, three objects as candidates for pairing are shown in the second image according to the distances between the objects. Four objects in the first image are shown. (d) Paired objects between the first and second image are depicted as particles in the same color. Arrows, three examples of paired objects. Landmarks are also depicted. The original images were 8‐bit images where the intensities of each particle correspond to the IDs of the objects, and the color was provided by setting lookup tables (ImageJ > Image > Lookup Tables > 3‐3‐2‐RGB)

We applied our method to mouse blastocysts. In Figure 2 (“*z*‐slice” and “MIP”), we chose nine nuclei as landmarks. In addition to the landmarks, we also labeled several tens of nuclei as objects of interest. We loaded the *xyz*‐coordinates of the landmarks into our ImageJ macro, and then computationally obtained the values of the three angles for 3D rotation. Note that the rotation centers in the two images were set at the centroids of the landmarks. Finally, we generated an image where the rotated positions of the landmarks were depicted as particles. This image is a 3D image (i.e., composed of multiple *z*‐slices). Figure 4(a) shows a merged 3D image of the landmarks from the first and second images. Before the rotation, the landmarks from the two images were not closely located (Figure 4(a), left panel), whereas after the rotations, they were (Figure 4(a), right panel).

Then, we applied the 3D rotation to the positions of the nuclei other than the landmarks, and generated a 3D image (i.e., composed of multiple *z*‐slices). In the 3D image where the nuclei from the first and the rotated second images were merged (Figure 4(b), right panel), most of the nuclei from the two images were closely located and paired each other (e.g., labeled by yellow). We can also find several nuclei which did not have counterparts (e.g., labeled by light blue with dashed line); this is because we failed to label all the nuclei at step 2 in Figure 3. In other words, the 3D visualization helped us to judge whether we had successfully labeled all nuclei of interest. In addition, we can also check the localization of the positions of the rotated nuclei in each *z*‐slice (Figure 4(b), left panel).

To determine the correspondence of each nucleus between the first and the rotated second images, we calculated the distances between the nuclei in the two images. For each nucleus in the first image, we searched for the nearest nucleus in the rotated second image. Figure 4(c) shows the ID of the nearest nucleus and the distance between the paired nuclei. We also searched for the second and third nearest nuclei, as shown in Figure 4(c). By quantitatively evaluating the distances of these three candidates, we can judge which nucleus is the counterpart of each nucleus from the first image. In the case that an object in the second image is multiply assigned as the nearest neighbor for different objects in the first image, such multiply assigned objects are also listed in the output text file (not shown in Figure 4(c)). Multiply assigned objects also emerge when the numbers of objects are inconsistent between the two images. Simultaneously, under the assumption that the nearest nucleus is the correct counterpart, we generated a 3D image where the paired nuclei were presented by the same color (Figure 4(d)).

### 3.2 3D reconstruction of rotated image

**FIGURE 5:**
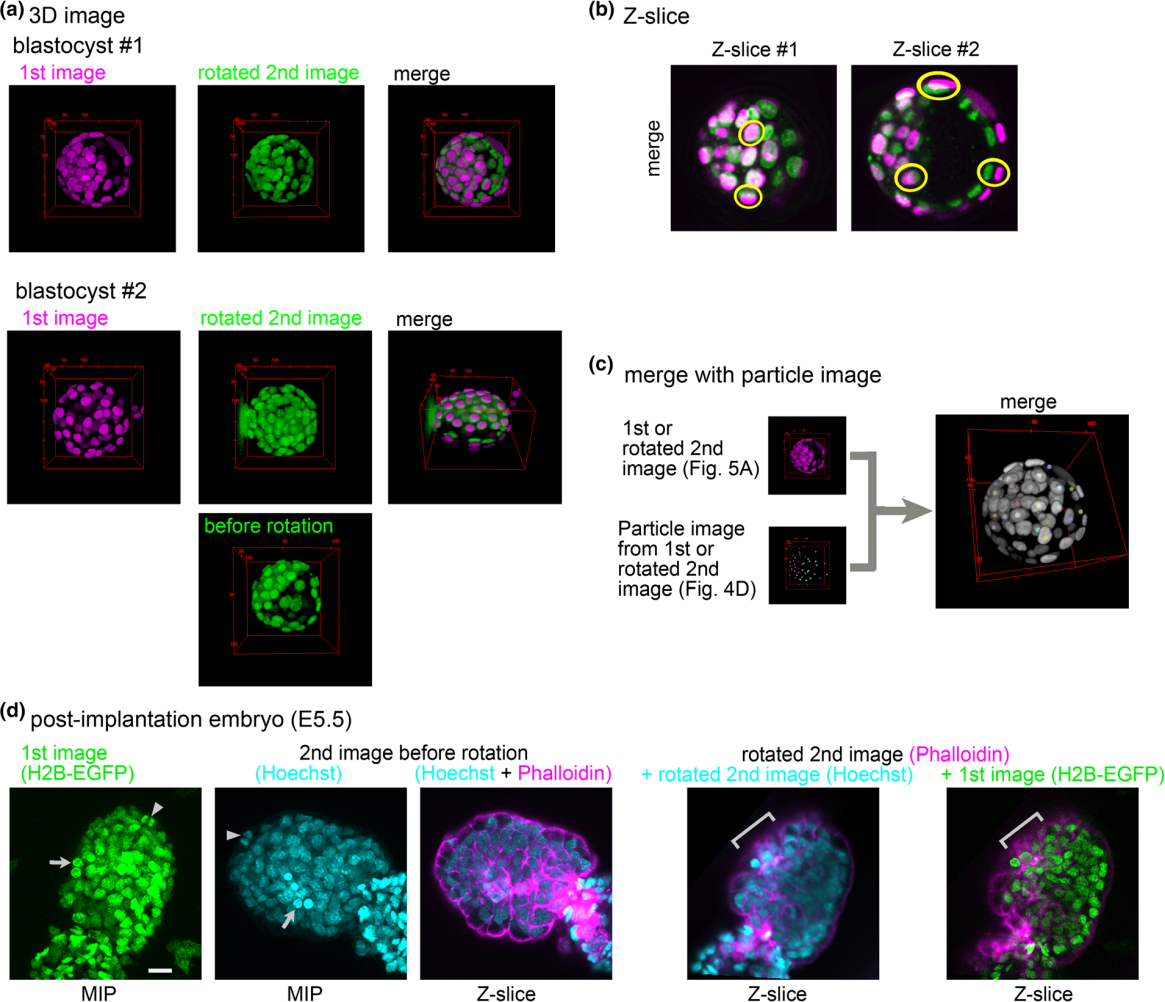
Three‐dimensional (3D) reconstruction of a rotated image. (a) 3D images of the first and the rotated second image are shown for two blastocysts (#1 and #2). The second images before rotation are shown in Figure 2 for #1 or in the bottom panel for #2. Note that before the rotation, the intensities of the second images were normalized along the *z*‐axis (ImageJ > Process > Enhance Contrast… > Normalize), and thus, the intensities were not conserved. (b) Two *z*‐slices of the merged image of blastocyst #1 are shown. Yellow, examples of paired nuclei between the first and the rotated second image. (c) A merged image is shown where images of nuclei can be the first or the rotated second image, and particle images constructed in Figure 4 can be the first or the rotated second image. In other words, four (2 × 2) combinations of merged images can be generated. The merged image was generated by ImageJ > Image > Color > Merge Channels… (d) Embryos at 5.5 days after fertilization (E5.5). The first to third images are before 3D registration, and the fourth and fifth images are after 3D registration. Arrows and arrowheads; examples of landmarks. The number of landmarks = 7. Around the regions indicated by gray bars in the fourth and fifth images, the phalloidin signal was well detected. Scale bars = 20 μm

We developed an algorithm to reconstruct rotated second images. We applied the above 3D rotation to the second image itself (i.e., pixel/voxel‐based rotation is applied). In Figure 5(a), the rotated second image is shown (blastocyst #1 vs. Figure 2, which are the images before the rotation). A merged image of the first and the rotated second images exhibited good correspondences of the nuclei. Moreover, the correspondences are also confirmed by analyzing the *z*‐slice images in Figure 5(b) (e.g., labeled by yellow). The slight spatial discrepancies between some pairs of nuclei may result from shrinkage of the blastocyst by the fixation process. Chromosome segregation was observed in the second image (Figure 5(b), at the upper right of *z*‐slice #2, which was stained by Hoechst), whereas the signal in the first image was obscure; this is because the nuclear localization signal, which does not bind to the chromosomes, was used in the first image. Another example of a blastocyst is shown in Figure 5(a) (blastocyst #2). Although the directions between the first and the second image before the rotation were quite different (first image vs. before rotation), the directions became absolutely aligned after the rotation (first image vs. rotated second image), and the merged image showed clear correspondences between the paired nuclei.

As an additional function in our tool, we can generate merged images between the nuclear images and the particle images (Figure 5(c)), which is useful to identify the IDs of the nuclei in the nuclear images.

To assess the applicability of our method to other tissues, we selected post‐implantation embryos at 5.5 days after fertilization. Specifically, we tried to perform 3D registration between F‐actin‐stained images and nuclear‐stained images. For embryos before fixation (first images in Figure 5(d)), we labeled the nuclei (Figure 5(d), first panel, H2B‐EGFP). For embryos after fixation (second images in Figure 5(d)), we stained F‐actin by phalloidin, and we simultaneously stained the nuclei by Hoechst for internal standards (Figure 5(d), second and third panels). In other words, we performed 3D registration by using the nuclei as landmarks from both embryos, and then we compared the nuclear positions with the phalloidin localization. The landmarks of the nuclei are illustrated in the two images in Figure 5(d) (Figure 5, arrows and arrowheads). After obtaining the optimal values of the three angles, we reconstructed 3D rotated images. In the rotated second image, the embryo exhibited a similar direction to that in the first image (Figure 5(d), the fourth panel vs. the first panel). The fifth panel in Figure 5(d) is a merged image between the nuclei in the first image and the F‐actin in the rotated second image. The F‐actin signal was correctly detected in the interspaces of the nuclei (indicated by gray bars in Figure 5(d)), which would correspond to cell–cell boundaries.

### 3.3 Performance and accuracy of our algorithm

**FIGURE 6:**
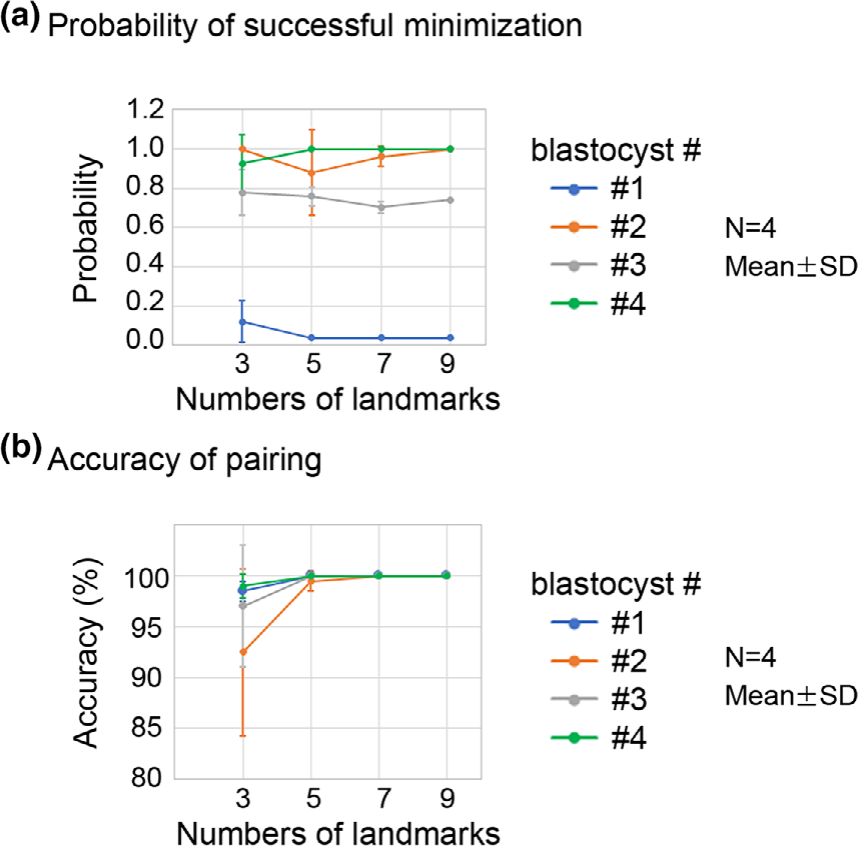
Performance and accuracy of 3D registration. (a) The performance of the minimization process was evaluated. The probability of successful minimization among 27 trials is shown for each blastocyst (#1–#4); probability = 1.0 means that the global minimum was successfully reached in all 27 trials. For number of landmarks = 9, *N* = 1. For number of landmarks = 3, 5, or 7, landmarks were randomly chosen from the nine landmarks, and four sets of different landmarks were generated; *N* = 4. (b) Accuracy of pairing of objects was evaluated for the outcomes of the successful minimization in (a). Similar to (a), the four blastocysts were tested with different numbers of landmarks for each blastocyst

We evaluated the performance of our algorithm. In our method, we searched for the optimal values of the three rotation angles as described previously. This kind of problem is called a minimization problem. In general, a minimization problem has a risk that the outcome is trapped at local minima of the cost function to be minimized, while the global minimum provides the optimal values. In our case, the cost function is the sum of distances between the paired landmarks as defined in Section 2. To reduce this risk, we performed multiple sets of minimizations in parallel from different initial values of the three rotation angles. For each angle, we set three initial values, resulting in 27 (3 × 3 × 3) sets of minimization processes. Some sets may reach the global minimum, while other sets may be trapped at local minima. In Figure 6(a), we calculated the probability of reaching the global minimum (i.e., the numbers of the trials reaching the global minimum among the 27 trials). In the case of nine landmarks, three blastocysts showed high probabilities (#2, #3, and #4), whereas one blastocyst showed a low probability (#1). Among the nine landmarks, we randomly selected three, five, or seven landmarks and performed the minimizations. For each blastocyst, the number of landmarks did not significantly affect the probability. These results suggest that the probability is largely dependent on individual blastocysts but not on the number of landmarks.

Next, we evaluated the accuracy of pairing the nuclei other than the landmarks. We manually defined the correct pairs of nuclei and examined whether the outcomes of the minimization are consistent with the correct pairs. Note that we only considered the outcomes of the global minimum. Figure 6(b) shows the percentage of correct pairs of nuclei. In the case of nine landmarks, all pairs obtained by the minimization were correct (i.e., accuracy = 100%). On the other hand, with smaller numbers of landmarks (three or five), the accuracy was reduced. Based on these observations and Figure 6(a), we think that the number of landmarks should be at least seven and that 27 sets of initial values of the three rotation angles are sufficient for most of samples.

### 3.4 Systematic evaluation of the accuracy of our algorithm

To further evaluate the accuracy of our method, we used artificially generated data with various situations possibly resembling real tissues. We considered (1) deformation of specimen/images and (2) positional noises of *xyz*‐coordinates of landmarks and objects of interest. Because our 3D registration method solely requires *xyz*‐coordinates of landmarks but not images, we did not explicitly present images.

**FIGURE 7:**
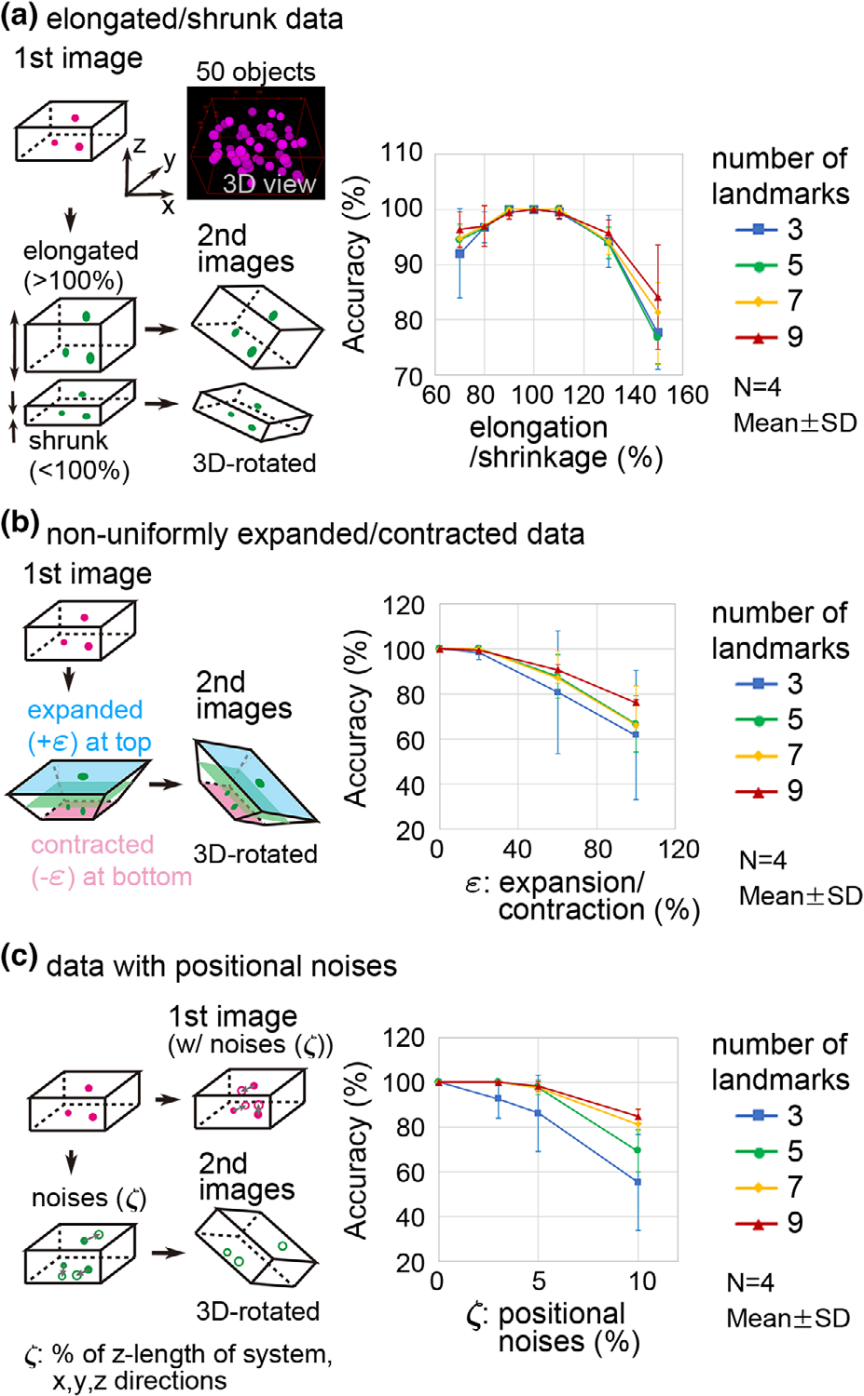
Accuracy of three‐dimensional (3D) registration in artificially generated data. (a) The second images were generated through elongation/shrinkage along the *z*‐axis. In the first images, the objects and landmarks were randomly distributed in a space with *x* = 0–200, *y* = 0–150, *z* = 0–100. Four independent first images were generated (*N* = 4) with subsequent generation of second images. The accuracy was calculated in a similar manner to Figure 6(b). (b) The second images were generated through non‐uniform expansion/contraction of *xy*‐planes in a manner dependent on the *z*‐position. The first images were identical to those in (a). The top *xy*‐plane was expanded (light blue); the bottom *xy*‐plane was contracted (magenta); the exact mid of the *xy*‐plane was not deformed (light green). (c) The second images were generated through addition of positional noise for each object and landmark. Similarly, positional noise was also added to the first images, which was different from the noise for the second images. The positional noise was randomly provided with uniform distributions (from –*ξ* to +*ξ*) along the three directions (*x*, *y*, *z*), and their magnitudes (*ξ*) are shown as relative values to the *z*‐length of the system (i.e., *z* = 0–100)

In Figure 7(a), we considered shrinkage/elongation of specimens/images. As the first images, we computationally set multiple objects with *xyz*‐coordinates randomly provided (Figure 7(a), “3D view” which were generated according to the *xyz*‐coordinates of the objects). As the second images, we shrank or elongated the first images along the *z*‐axis, resulting in the positional modification of the *z*‐coordinates of the objects. Then, we 3D rotated the images with the objects (Figure 7(a), left panel). Among the 50 objects, we randomly selected three to nine landmarks, and then we applied our method. The right panel of Figure 7(a) shows the accuracy of pairing objects under various shrinkage/elongation conditions. Under no shrinkage/elongation (i.e., shrinkage/elongation = 100%), the accuracy was 100% even in the case of three landmarks. When shrinkage (<100%) or elongation (>100%) was applied, the accuracies were decreased. Overall, with deformation ranging from 80% to 120%, our method showed high accuracy (>~95%). Basically, we found a trend that increasing the number of landmarks improved the accuracy (e.g., three landmarks vs. nine landmarks).

In Figure 7(b), we considered non‐uniform deformation of images. The first images with the objects were identical to those in Figure 7(a). By contrast, as the second images, we expanded or contracted the *xy*‐plane in a manner dependent on *z*‐position (Figure 7(b), left panel): The *xy*‐plane was expanded at the top position (Figure 7(b), left panel, light blue plane) and contracted at the bottom position (Figure 7(b), left panel, magenta plane). We denote the rate of expansion/contraction as *ε*. For instance, if *ε* = 50%, the lengths of both *x* and *y* showed a 50% increase or decrease at the top or bottom position, respectively. At the intermediate positions, the rates were linearly interpolated, resulting in a trapezoid‐like deformation. Similar to Figure 7(a), we randomly selected landmarks and applied our method. The accuracy was 100% under no deformation conditions (Figure 7(b), right panel, *ε* = 0%), whereas the accuracy decreased under deformation (*ε* > 0%). Under the conditions of *ε* < 20%, our method showed high accuracy. Similar to Figure 7(a), increasing the number of landmarks improved the accuracy.

In Figure 7(c), we considered positional noises of *xyz*‐coordinates of both landmarks and objects. We provided positional noise for landmarks and objects in both first and second images. The magnitudes of the positional noise were ±*ξ* (%) relative to the *z*‐length of the system for each axis (*x*, *y*, and *z*). The accuracy was 100% under no positional noise (*ξ* = 0), whereas the accuracy decreased under increased noise. Under the conditions of *ξ* < 5%, our method showed high accuracy. Similar to Figure 7(a,b), increasing the number of landmarks improved the accuracy.

## 4 DISCUSSION

In the present study, we developed a landmark‐based 3D registration tool with subsequent 3D image reconstruction, and demonstrated that this tool works well for mouse blastocysts composed of several tens of nuclei and mouse post‐implantation embryos. This tool includes several ways of visualizing and quantifying the registration outcomes, which enables us to objectively judge which nucleus in one image corresponds to a certain nucleus in another image. Importantly, to ensure versatility in the field of experimental biology, this tool can run as ImageJ macros.

### 4.1 Applicability to objects other than nuclei

We chose nuclei as landmarks, but any object is permitted. A sole requirement of our tool is that users can identify the same position in the first and second images. Therefore, even in the case that different markers are used between the two images, we can perform 3D registration if landmarks are correctly defined. Similarly, objects of interest (Figure 3, step 2) are not limited to nuclei. In addition, even if we do not choose any objects of interest, we can carry out 3D image reconstruction using landmarks. These flexibilities of our tool expand its range of applicability.

### 4.2 Accuracy of our method

The accuracy of our 3D registration was evaluated (Figures 6 and 7). In principle, our registration method requires at least three landmarks: One or two landmarks are not enough to uniquely determine 3D positions of other objects due to a geometric reason, resulting in unsuccessful 3D registration. In addition, landmarks should be not linearly arranged in 3D space; in the case of three landmarks, they should exhibit a triangular arrangement to uniquely determine 3D positions of other objects. Under the ideal conditions where the *xyz*‐coordinates of all landmarks (at least three) and objects are precisely provided (i.e., no positional noise and no deformation of specimens/images), 3D registration should be perfect and the accuracy also becomes 100% in principle, as proven in Figure 7. However, in real specimens, positional errors of the *xyz*‐coordinates are not avoidable due to many factors including sample preparation procedures, microscopy settings, and labeling procedures of landmarks/objects. Therefore, under the conditions of three landmarks in our analyses, the accuracy did not reach 100% (Figure 6(b)). By increasing the number of landmarks, the accuracy was improved (Figures 6 and 7(a)–(c)). This is because the positional errors of the multiple landmarks are averaged and hence the undesired effects of the errors are canceled out, given that the positional noise is random (e.g., Gaussian distribution in 3D space). In our analyses using blastocysts, although we did not do anything special to increase the preciseness except that we roughly selected positions equivalent to the 3D centroids of the nuclei, seven or nine landmarks were enough to achieve high accuracy. In summary, to achieve high accuracy, we recommend that users improve the preciseness of the *xyz*‐coordinates where procedures prior to the image processing are also included, while users should increase the number of landmarks to cancel out the effects of positional errors.

### 4.3 Comparison with other possible methods

Here we discuss the comparison of our method with other 3D registration methods. Except for the methods described in the Introduction section, another possible method is as follows. The most straightforward strategy of 3D registration is based on pixel‐by‐pixel correlation of intensities between two images. By translating and rotating one of the two images, we can search for the image transformation which gives the highest correlation. In order for this method to work well, some requirements should be met. For instance, decay of intensities along the *z*‐direction should be slight, because decay significantly affects the value of the correlation. However, in real images of biological specimens, intensities usually decay along the *z*‐direction. Another requirement is related to image qualities, but we cannot expect comparable qualities between images from live specimens and from fixed specimens. A more critical point is its limited applicability to cases where two specimens are stained by different markers showing different localizations: nucleus vs. cytoplasm, something fluorescently labeled vs. micro‐CT, etc. In these cases, the correlation of intensities between the two images is meaningless. From the viewpoint of computational load, the search for the highest correlation in 3D translation and rotation may result in unrealistic running time. To overcome these issues, block matching algorithms have been developed, where each block contains many pixels/voxels and the correlation between the blocks from two images is evaluated. A practical tool based on this algorithm is available as a Fiji plugin, which was used for different types of images derived from relatively large tissues (mm–cm) (Fernandez & Moisy, 2021). Because the tool is for 3D image reconstruction, correspondences between objects of interest such as nuclei are not evaluated.

Our method is based on the manual choice of landmarks. Although this strategy is primitive, we can potentially label correct landmarks under the situations raised in the previous paragraph; information of tissue geometries, or even noises derived from intrinsic fluorescence of something like chloroplasts, etc., can be used to set landmarks. Tissue geometries were actually used as landmarks for 3D registration of *Drosophila* brains (Peng et al., 2011). In the case that users want to align non‐particulate structures such as filaments, one possible solution is simultaneous imaging of nuclei for usage as landmarks, as we showed in Figure 5(d). We emphasize, “use any signal/information available for landmarks.” Therefore, we think that the manual selection of landmarks expands the applicability of our method.

### 4.4 Expertise required to implement the method

Our method was developed as ImageJ/Fiji's macros (note that we recommend Fiji). For computers where Fiji is installed, the macros can run immediately after downloading them (i.e., no additional setting). Usual laptops are sufficient to run the macros (e.g., MacBook Air). We provide the protocols with the macros on the Web site described in Supporting information.

## CONFLICT OF INTEREST

The authors declare no competing financial interests.

## Supporting information


**Data S1.** Supporting information

## APPENDIX A

### A.1 Definition of 3D rotation

In the case of 2D situations, the rotation is defined as the following matrix: $\left(\begin{array}{c} {x_{i}}^{\prime }\\ {y_{i}}^{\prime } \end{array}\right)=\left(\begin{array}{cc} \cos\theta & -\sin\theta \\ \sin\theta & \cos\theta \end{array}\right)\left(\begin{array}{c} x_{i}\\ y_{i} \end{array}\right),$

where *θ* is the rotation angle, *x* and *y* are the original coordinates, *x*′ and *y*′ are the coordinates after rotation, and *i* is the position of the *i*th object, such as nuclei and pixels. In the case of 3D situations, the rotation matrix is defined as follows:

$$\left(\begin{array}{c} {x_{i}}^{\prime }\\ {y_{i}}^{\prime }\\ {z_{i}}^{\prime }\\ 1 \end{array}\right)=\left(\begin{array}{cccc} \cos\phi \cos\theta & \cos\phi \sin\theta \sin\psi -\sin\phi \cos\psi & \cos\phi \sin\theta \cos\psi +\sin\phi \sin\psi & 0\\ \sin\phi \cos\theta & \sin\phi \sin\theta \sin\psi +\cos\phi \cos\psi & \sin\phi \sin\theta \cos\psi -\cos\phi \sin\psi & 0\\ -\sin\theta & \cos\theta \sin\psi & \cos\theta \cos\psi & 0\\ 0 & 0 & 0 & 1 \end{array}\right)\left(\begin{array}{c} x_{i}\\ y_{i}\\ z_{i}\\ 1 \end{array}\right),$$

where *ϕ*, *θ*, and *ψ* are the angles expressing the 3D rotations.

### A.2 Cost function to be minimized

To fit the *xyz*‐coordinates of landmarks from a second image to those from a first image by 3D rotation, we considered the summation of the distances between paired landmarks from the two images as follows:

$$G=\sum_{i=1}^{I}\left\{{\left({x_{i}}^{\prime }-X_{i}\right)}^{2}+{\left({y_{i}}^{\prime }-Y_{i}\right)}^{2}+{\left({z_{i}}^{\prime }-Z_{i}\right)}^{2}\right\},$$

where *G* is the sum, *I* is the total number of landmarks, and *X*, *Y*, and *Z* are the *xyz*‐coordinates of the landmarks from the first image. We searched for the values of *ϕ*, *θ*, and *ψ* which minimized the value of *G*, and thus, *G* is the cost function to be minimized.

### A.3 Minimization procedure

The minimization of *G* was achieved using a Monte Carlo algorithm. Initially, *ϕ*, *θ*, and *ψ* were set to be certain values: In the case of Figure 6, where 27 sets of initial values were provided, the values of each angle were 0, (2/3)*π*, or (4/3)*π*. These values were iteratively modified so that the value of *G* became smaller, and consequently, *G* was expected to be minimized (i.e., reaching the global minimum) unless the process was trapped at a local minimum.

More precisely, our algorithm was based on a Markov chain Monte Carlo (MCMC) method as follows. For each iterative process, a slight modification of the values of the three angles was applied. If the value of *G* became smaller compared with the value before applying the above modification, the modified values of the angles were accepted. On the other hand, if the value of *G* became larger, the modified values of the angles were rejected with a certain probability. Parameters related to the probability may be set by users; parameter conditions providing high probability increase the risk that the process is trapped at local minima, whereas parameter conditions providing low probability increase the risk that the value of *G* does not become sufficiently small. We set the parameter values sufficiently good for our samples. The minimization procedure was implemented as a macro of ImageJ (Macro_3D_particle_registration_06_v2.ijm). The version of Fiji we used was 1.53. The running time in the case of nine landmarks was ~50 s on our laptop (MacBook Air, CPU: Core i7, Dual core, 1.7 GHz; memory: 8 GB 1600 MHz DDR3). See the description in the macro code for practical information.

### A.4 3D depiction of landmarks and objects of interest

According to the *xyz*‐coordinates of landmarks and objects of interest, they were depicted as particles in the 3D image (i.e., multiple *z*‐slices) (Figure 4). This was implemented as a macro of ImageJ (Macro_particle_drawing_02.ijm). The radius of the particles can be set by users. The running time was several sec at the most (MacBook Air). See the description in the macro code for practical information.

### A.5 3D image reconstruction

When we reconstructed 3D rotated images, we applied the values of the three angles to the *xyz*‐coordinates of each pixel/voxel. The intensities of the rotated voxels are averaged by a mean filter of a 3 × 3 × 3 kernel. This was implemented as a macro of ImageJ (Macro_3D_image_rotation_02.ijm). In the case of image size = (262 (*x*) × 262 (*y*) × 165 (*z*) pixels), the running time was ~15 min (MacBook Air). In the case that the mean filter of a 3 × 3 × 3 kernel was not applied, the running time was ~2 min, but the outcome image was very noisy; the *z*‐resolutions of input images (i.e., widths of *z*‐slices) should largely affect the quality of outcome images. Theoretically, if the images are 2‐fold larger along the *x*‐, *y*‐, and *z*‐axes, the running time may increase ~2^3^ = 8‐fold. See the description in the macro code for practical information.

### A.6 Correction of distortion of *xyz*‐coordinates

Step 3 in Figure 3 was implemented in the above macro for the minimization procedure (Macro_3D_particle_registration_06_v2.ijm). Shrinkage/elongation of each axis can be set in the macro. The detailed manual is provided on the Web site described in Supporting Information.

### A.7 Setting of landmarks and objects of interest in the blastocysts

We manually labeled the landmarks and the objects of interest by using a basic ImageJ function called Multi‐point tool. We selected a point which was roughly equivalent to a centroid of the nucleus where we considered not only the *x*‐ and *y*‐axes but also the *z*‐axis (e.g., for the *z*‐axis, we visually selected a *z*‐slice close to the centroid of the nucleus). Any other tools or procedures can be adopted by which the *xyz*‐coordinates are obtained.
