# Supplementary material for: An ImageJ‐based tool for three‐dimensional registration between different types of microscopic images
Source: Dev Growth Differ. 2023 Jan 14;65(1):65–74. doi: 10.1111/dgd.12835 (PMC10107647; doi:10.1111/dgd.12835)
Supplement: Supplementary file 1 — Data S1. Supporting information [file DGD-65-65-s001.docx]

The ImageJ macros and sample files are uploaded with the protocol on Github (<https://github.com/hfkoyama/3D_registration>) and figshare ([https://doi.org/10.6084/m9.figshare.21485547](http://dx.doi.org/10.6084/m9.figshare.21485547)).
